# Dietary diversity of 6- to 59-month-old children in rural areas of Moramanga and Morondava districts, Madagascar

**DOI:** 10.1371/journal.pone.0200235

**Published:** 2018-07-13

**Authors:** Nivo Heritiana Rakotonirainy, Valérie Razafindratovo, Chitale Rabaoarisoa Remonja, Randza Rasoloarijaona, Patrice Piola, Charlotte Raharintsoa, Rindra Vatosoa Randremanana

**Affiliations:** 1 Epidemiology unit, Institut Pasteur de Madagascar, Antananarivo, Madagascar; 2 Laboratoire de Biochimie Appliquée aux Sciences de l’Alimentation et à la Nutrition, Faculté des Sciences, Université d’Antananarivo, Antananarivo, Madagascar; 3 Epidemiology and Public Health Unit, Institut Pasteur du Cambodge, Phnom Penh, Royaume du Cambodge; Zhejiang University College of Biosystems Engineering and Food Science, CHINA

## Abstract

**Background:**

A dietary imbalance or a disregard for the nutritional needs of children during early childhood can affect their growth. From the age of six months, breast milk is no longer able to meet the energy and micronutrient needs of children; the consumption of adequate complementary foods is therefore essential. Various indicators have been used to assess the quality of children's diets, and the dietary diversity score is a good indicator of children's diets. The objective of this study was to describe the dietary practices of children in rural areas of Moramanga and Morondava, Madagascar, and to identify the determinants of low dietary diversity to prioritize nutritional interventions.

**Methods:**

We collected dietary data in 2014 on children aged 6–59 months in a study on the determinants of chronic malnutrition using the 24-hour recall method. Data on the characteristics of households and mothers were also collected. We carried out bivariate and multivariate analyses to identify the determinants of low dietary diversity scores for children.

**Results:**

We included 1824 children: 893 from Moramanga and 931 from Morondava. Approximately 42.1% [95% CI: 39.0–45.4] of the children from Moramanga and 47.6% [95% CI: 44.4–50.8] of those from Morondava had a poorly diversified diet, consisting mainly of foods rich in carbohydrates and poor in meat products. Poor maternal education was associated with a high likelihood of having a non-varied diet in both study areas; the adjusted odds ratios were 2.2 [95% CI: 1.3–3.8] and 4.0 [95% CI: 2.5–6.4] for children from mothers with lower education levels for Moramanga and Morondava, respectively. For children recruited in Morondava, having low household socioeconomic status (adjusted OR: 1.8, 95% CI: 1.2–2.8) and belonging to a household without livestock was associated with a low dietary diversity score (adjusted OR: 1.8, 95% CI 1.2–2.7).

**Conclusion:**

Our results show the need to improve girls' education, adapt nutrition education programs for mothers based on their level of education, and strengthen poverty reduction programs.

## Introduction

Malnutrition remains a serious problem in most developing countries, affecting particularly vulnerable groups, especially children under the age of five. It is directly or indirectly responsible for 45% of all child deaths worldwide [1]. Moreover, malnutrition affects the psychomotor development and learning capacities of children, with a long-term impact on the socioeconomic development of the country. Malnutrition occurs due to the interaction of several determinants at different levels: the community, the household, and the individual. At the individual level, malnutrition results from inadequate diets that do not provide sufficient calories and micronutrients, from clinical or sub-clinical infections, or from a combination of both [2]. From the age of six months, breast milk is no longer able to meet the energy and micronutrient needs of children; the consumption of adequate complementary foods, in quality and quantity, is therefore essential [3]. Various indicators have been used to assess the quality of children's diets. The dietary diversity score is a good indicator of children's diets [4–6] and can be used to assess macro- and micronutrient consumption [7,8].

Appropriate feeding practices are insufficiently widespread in Madagascar: 41.9% of children aged 6–23 months are exclusively breastfed, and only 30.9% receive dietary supplementation [9]. Malnutrition is one of the main causes of the high rate of infant and child mortality (62‰ in 2012) [9]. The prevalence of chronic malnutrition is 47.4% and that of acute malnutrition is 8% [9].

The objective of this study was to describe the diet of 6- to 59-month-old children in two districts of Madagascar (Moramanga and Morondava) and to identify the determinants of low dietary diversity scores. These findings are essential for improving or adapting interventions concerning children's diets to improve their nutritional status.

## Materials and methods

### Study sites and population

The children included in this study on feeding practices were those participating in a study on the determinants of chronic malnutrition [10], for which data collection took place from February to November 2014. The study was carried out in two rural areas, the Health and Demographic Surveillance Site (HDSS) of Moramanga and the Bemanonga Commune of the Morondava district. These two areas were selected, as they belong to 2 regions with different nutritional profiles: the Alaotra-Mangoro region is an area of high stunting prevalence (50–60%), whereas the Menabe region is located in an area with average stunting prevalence.

The HDSS site of Moramanga is in the middle-eastern part of Madagascar in the Alaotra-Mangoro region and has 70,000 inhabitants. It is divided into three communes composed of 30 fokontany (villages). A survey conducted in 2010 showed that this area had a high prevalence of chronic malnutrition, estimated at 59.6% [9]. The district of Moramanga has a hot and humid tropical climate, an annual average rainfall of 1500 to 2000 mm, and light and frequent rainfalls, even in the winter. Staple crops (tubers, cereals) and crops of fruits and vegetables are common, as is the raising of poultry, goats, and pigs. The region of Alaotra-Mangoro is rich in mineral resources. The Moramanga district has the most mines (47.6% of its municipalities have mining operations) and is home to one of the country's largest mining operations (cobalt and nickel) in the Ambatovy zone [11].

The study involved 13 villages in the rural community of Bemanonga in the Morondava district of Menabe. These 13 villages have an estimated population of 13,700 inhabitants (9% of the total population of the district of Morondava). The district of Morondava is located in the southwestern region of Madagascar. It has very low and irregular rainfall (< 800 mm per year), with six to eight months per year of the dry season. Farmers in the district mainly grow rice but also other crops, such as cassava, maize, and peas. The raising of zebu is common in the district, and families practice poultry farming [12].

The children were chosen randomly from the two study areas. First, a screening phase was conducted by measuring weight, height/length of all children under 5 years of age. Thereafter, simple random sampling of non-malnourished and stunted children was performed until the number of children included was at least 810 in each study site. If mothers had more than one eligible child, only the first randomly selected child was considered. All children under 5 years with disabilities preventing anthropometric measurements were not included in the screening phase. Those with both stunting and wasting and those not accompanied by their own mothers or caregivers during the survey were not included in study [10].

The parents of the children included in the study gave their written consent after being given information on the purpose and practical issues of the study. The protocol for the study was approved by the Ethics Committee of the Ministry of Public Health of Madagascar (N°042-MSANP/CE of 13/06/2014).

### Data collection

The questionnaire was administered to the mother, or the person who usually takes care of the child at home, by trained interviewers. Information was collected on the following characteristics: (i) the child’s age and sex; (ii) feeding practices, including breastfeeding, age at introduction of supplementary food, consumption of food and beverages, and meal frequency using the 24-hour recall method; (iii) the mother’s age, education, and occupation; and iv) regarding the household, property owned, the characteristics of the house, ownership of livestock, and the number of food crops cultivated (rice, fruits, tubers, legumes, vegetables, and others).

### Data analysis

#### Evaluation of the feeding practices of children 6 to 23 months of age

The information collected on diet was used to develop several indicators of dietary practices for children aged 6 to 23 months. We considered the standard indicators proposed by the WHO [13]: early breastfeeding, breastfeeding for up to 12 months, consumption of supplementary food, minimum meal frequency, minimum acceptable diet, and consumption of iron-rich foods. The proportion of children with minimum acceptable diets is defined as the proportion of breastfed and non-breastfed children aged 6–23 months who i) received solid, semisolid, or soft foods (including milk feeds for non-breastfed children) the minimum number of 2 and 3 times for breastfed infants and 4 times for non-breastfed infants and ii) have consumed foods from at least 4 of the following food groups: starchy staples (grains, roots and tubers), legumes and nuts, dairy products, flesh foods, eggs, vitamin A-rich fruits and vegetables, and other fruits and vegetables.

Iron-rich foods include flesh foods, such as meat, poultry, fish, and liver/organ meat.

#### Estimation of the food diversity score

Food consumed by children the day before the survey was classified into the following seven food groups according to WHO guidance: (1) cereals, roots and tubers; (2) legumes and nuts; (3) milk and its derivatives; (4) meat products (meat, poultry, offal, and fish); (5) eggs; (6) vitamin A-rich fruits and vegetables (leafy green vegetables, yellow fruits and vegetables); and (7) other fruits and vegetables [13]. The dietary diversity score (DDS) was defined as the number of food groups consumed by the child the previous day. A DDS of four is considered the minimum DDS. Accordingly, a child with a DDS < 4 was classified as having low dietary diversity; otherwise, he was considered to have adequate dietary diversity [13].

Data collected about the household, apart from the possession of livestock and the number of food crops cultivated, were used to determine the household’s socioeconomic level. The wealth index was constructed using principal component analysis (PCA) for continuous variables and multiple correspondence analysis (MCA) for categorical variables. The variables included in the generation of this score were those having a dominant modality with a frequency of less than 80% (household assets, including radio, cart, television, bicycle; number of inhabitants per room; materials used for the roof and walls; combustible used for light and cooking; type of toilet; ownership of locales for cooking and bathing; source of drinking water; and the distance from the house to the source). The coefficients of each linear combination were used as a weight for each variable.

We defined four wealth index levels by treating the distribution quartiles as the interval threshold, with the 1st quartile representing the poorest segment of the population and the 4th quartile representing the wealthiest. Statistical analysis of the data was performed using R software (R Development Core Team (2008). R Foundation for Statistical Computing, Vienna, Austria)

Qualitative variables are presented as percentages rounded to one decimal and quantitative variables as medians with interquartile ranges. The proportions were compared using a X^2^ test, a Yates-adjusted X^2^ test, or Fisher’s exact test. Averages and medians were compared using a means or median comparison test.

Bivariate analysis was used to identify the explanatory variables to be included in the multivariate analysis. The variable dietary diversity score was classified into two categories: low and adequate. Explanatory variables with a p-value < 0.2 were included in the multivariate analysis. A backward logistic regression was performed to obtain the final model and the variables associated with a low dietary diversity score.

## Results

### Characteristics of the children included in the study

We included 1824 children in the study: 893 from Moramanga and 931 from Morondava.

In Moramanga, 7436 children under 5 years of age participated in the anthropometric measurements, 7359 (99.0%) of whom had valid measurements. For the study, we randomly selected 7139 children without wasting. In Morondava, 2048 children were screened, 1971 (96.2%) of whom had valid measurements; 1888 children were randomly selected for the study. The characteristics of the children are summarized in Table 1. Most were aged 24 months and over: 67.0% and 63.3% in Moramanga and Morondava, respectively. Among the children enrolled in the study, 52.7% in Moramanga and 49.0% in Morondava were female. There was a significant difference between the two study areas for all characteristics studied, except the sex of the children and the socioeconomic level of the households (Table 1).

**Table 1:** Characteristics of the children included in the study.

Characteristics	Moramanga N (%)	Morondava N (%)
**Child**		
Age		
6–11 months	69 (7.7)	107 (11.5)**
12–23 months	226 (25.3)	234 (25.1)
24–35 months	192 (21.5)	229 (24.6)
≥ 36 months	406 (45.5)	361 (38.8)
**Sex**		
Female	470 (52.7)	457 (49.1)
Male	423 (47.3)	474 (50.9)
**Dietary diversity score (DDS)**		
Low DDS	376 (42.2)	444 (47.7)**
Adequate DDS	517 (57.8)	487 (52.3)
**Breastfeeding**		
Yes	290 (32.4)	258 (27.7)*
No	603 (67.6)	673 (62.3)
**Mother**		
**Age**: median [interquartile range]	29 years [24–36]	26 years [20–35]**
**Education level**		
No school	98 (11.0)	363 (39.0)**
Elementary school	534 (59.8)	435 (46.7)
Middle school or higher	261 (29.2)	133 (14.3)
**Activities outside the home**		
Yes	789 (88.4)	716 (77.0)**
No	104 (11.6)	215 (33.0)
**Household**		
**Wealth index**		
Quartile 1 (poorest)	237 (26.5)	259 (27.8)
Quartile 2	210 (23.5)	230 (24.7)
Quartile 3	223 (25.0)	220 (23.6)
Quartile 4 (wealthiest)	223 (25.0)	222 (23.9)
**Types of food crops**		
None	29 (3.2%)	56 (6.0%)**
1 to 3	394 (44.1%)	637 (68.4%)
4 or more	470 (52.7%)	238 (25.6%)
**Possession of livestock**		
Yes	715 (80.0)	804 (86.3)**
No	178 (20.0)	127 (13.7)
**Total**	**893**	**931**

*p < 0.05;

**p < 0.001;

### Quality of dietary practices of children of 6 to 24 months

In Moramanga, the prevalence of early initiation of breastfeeding (within 1 hour of birth) was 53.5%. In Morondava, approximately 24.0% of children were breastfed within 1 hour of birth. The practice of breastfeeding up to the age of 12 months was very frequent in the two study sites: 98.5% of children from Moramanga and 87.6% from Morondava were still being breastfed on the day before the survey.

All children in the study aged 6 to 9 months had already begun to consume complementary foods. Almost all children under 18 months of age (95%) had adequate meals according to their age and breastfeeding status, although the proportion decreased starting from 18 months of age (90.8% in Moramanga and 75.2% in Morondava).

**Fig 1:**
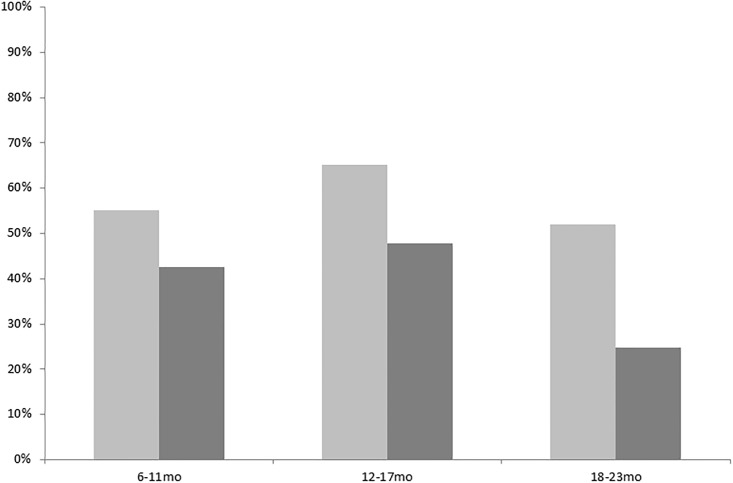
Minimal acceptable diet depending on age in the two study areas. Light gray bar: Moramanga, Dark gray bar: Morondava. Title of x axis: Age group (months). Title of y axis: Proportion of children with an acceptable minimum diet.

Overall, approximately 50% of children in both sites had an acceptable minimum diet. The proportion was highest for children aged 12 to 17 months: 65.1% in Moramanga and 47.8% in Morondava (Fig 1). It decreased from the age of 18 months. The decrease was most striking in Morondava, where only 24.8% of children aged 18–23 months had an acceptable minimum diet (Fig 1).

Iron-rich foods were eaten by 48.1% of children in Moramanga and 57.3% of those in Morondava. Children aged 12 to 17 months consumed the most iron-rich foods, 54.7% in Moramanga and 63.5% in Morondava. The proportion of children who consumed iron-rich foods was higher in Morondava than in Moramanga. The most highly consumed iron-rich foods were beef in Moramanga and fresh fish in Morondava.

### Food consumption

A median of eight foods were consumed (solid and semi-liquid) the day before the survey (interquartile range: 6–10). In Moramanga, half of the children consumed at least nine foods (interquartile range: 7–11), whereas in Morondava, the median number of foods consumed the day before was seven (interquartile interval: 5–9).

**Fig 2:**
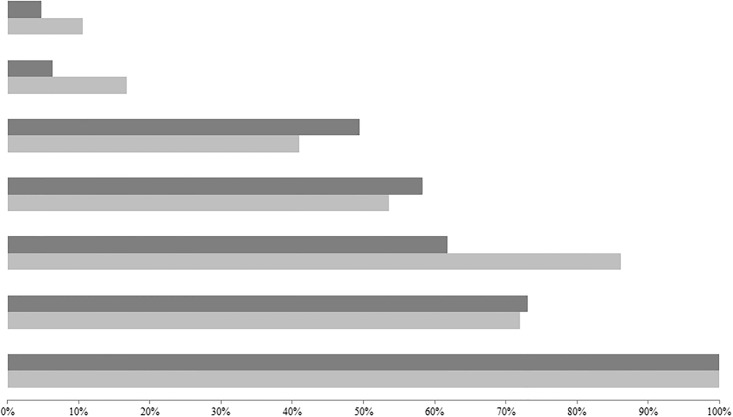
Frequency of consumption of the food groups in the two study areas. Light gray bar: Moramanga. Dark gray bar: Morondava. First two bars: Eggs. Second two bars: Dairy. Third two bars: Legumes and nuts. Fourth two bars: Meat, poultry and fish. Fifth two bars: Other fruits and vegetables. Sixth two bars: Vitamin A-rich fruits and vegetables. Seventh two bars: Cereals, roots and tubers.

The consumption frequencies of food groups in the two districts are summarized in Fig 2. The children's diet was composed mainly of "cereals, roots and tubers", "fruits and vegetables" (vitamin A-rich fruits and vegetables and other fruits and vegetables), and "meat products and fish".

These food groups were consumed by more than 45% of the children in the two districts. The consumption of eggs, and milk and its derivatives was rare, and less than 20% of children did so: 10.6% of children in Moramanga consumed eggs, and 16.8% consumed milk and dairy; in Morondava, 4.7% and 6.3% of children consumed eggs and milk and dairy, respectively.

The proportion of children who consumed food from groups other than staple foods (cereals, roots and tubers) and meat products was significantly higher in Moramanga compared to Morondava. Among those who ate meat products the day before, 53.1% came from Morondava and 46.8% from Moramanga (p < 0.05).

Rice was the staple food most frequently consumed by children at both study sites the day before the survey. Almost half (47%) of the children in Moramanga ate a cookie the day before and 41.4% of those from Morondava consumed sweet potato. In Morondava, children had consumed more vitamin A-rich fruits the day before, such as mangoes (64.3%), whereas in Moramanga, the most highly consumed fruit the day before was bananas (45.6%), which belong to the category of other fruit. The most popular vitamin A-rich vegetables at the two study sites were green leafy vegetables (Chinese cabbage, sweet potato leaf, etc.): 66.8% for Moramanga and 42.2% for Morondava. Various meats were consumed by the children of Moramanga: 25.3% beef, 18.6% dried fish, and 14.9% poultry. In contrast, more than half of the children (56.4%) in Morondava consumed fresh fish the day before, and 11.2% consumed beef and 8.3% dried fish. Concerning legumes/nuts/seeds, children in Moramanga consumed beans (44.3%) and peanuts (31.7%). In Morondava, 52.1% of children consumed lima beans/butter beans and 32.8% peanuts the day before.

### Dietary diversity and determinants

A low dietary diversity score was defined as the consumption of three or fewer food groups. The proportion of children with a low dietary diversity score was 42.1% [95% CI: 39.0%-45.4%] in Moramanga and 47.6% [95% CI: 44.4%-50.8%] in Morondava.

Children at the two study sites who had a low dietary diversity score rarely consumed eggs or foods belonging to the milk and its derivatives group. In Moramanga, less than 10% of children with a low dietary diversity score consumed dairy products and eggs, respectively at 7.0% and 9.5%. In Morondava, the proportion of children with a low dietary diversity score consuming dairy products the previous day was 8.5%, and the proportion of who consumed eggs was 4.5%.

**Fig 3:**
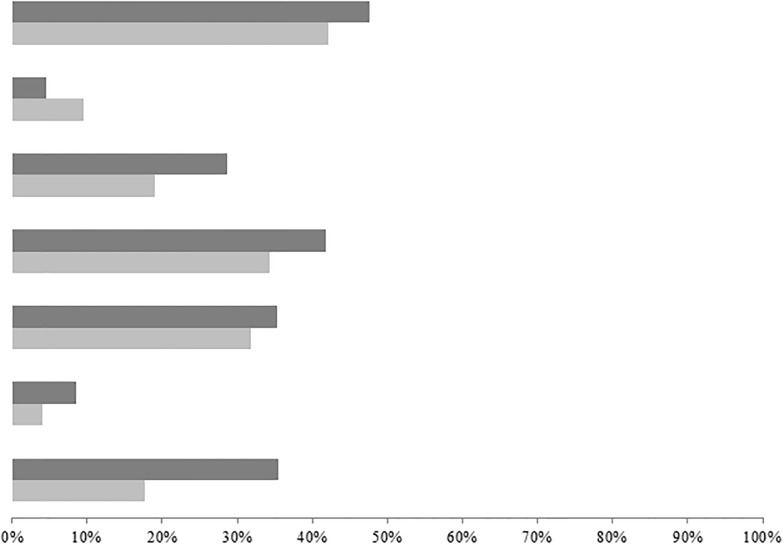
Proportion of children with a low dietary diversity score who consumed each food group. Light gray bar: Moramanga Dark gray bar: Morondava First two bars: Cereals, roots and tubers Second two bars: Eggs Third two bars: Meat, poultry and fish Fourth two bars: Other vegetables and fruits Fifth two bars: Vitamin A-rich fruits and vegetables Sixth two bars: Dairy Seventh two bars: Legumes and nuts.

Children in Morondava who had a low dietary diversity score mostly consumed foods belonging to the groups "legumes and nuts" and "meat products" (p < 0.001) (Fig 3).

We assessed the association between a low dietary diversity and many independent variables: household characteristics (wealth index, the ownership of livestock and the types of food crops), and mothers’ characteristics (age, occupation status and level of education). The final model showed that the main variables significantly associated with low dietary diversity were mothers’ education at both study sites and wealth index and non-possession of livestock in Morondava. In Moramanga, a low dietary diversity score was significantly associated with a low level of maternal education. Children whose mothers never attended school and those whose mothers completed at least one year in primary school were, respectively, 2.2-fold (95% CI: 1.3–3.8) and 1.8-fold (95% CI: 1.3–2.6) more likely to have a low dietary diversity score than those whose mothers spent at least one year in middle school or university (Table 2).

**Table 2:** Results of the multivariate analysis between the dietary diversity score and the explanatory variables, Moramanga district: Final model.

	Dietary diversity score		Crude OR (95% CI)	Adjusted OR * (95% CI)
	Low N (%)	Adequate N (%)		
Age (in months)				
≥36	151 (37.3)	255 (62.7)	1	1
24–35	78 (40.6)	114 (59.4)	1.1 (0.8–1.6)	1.1 (0.8–1.6
12–23	102 (45.1)	124 (54.9)	1.3 (0.9–1.8)	1.3 (0.9–1.8)
6–11	45 (65.2)	24 (34.8)	3.3 (1.9–5.8)	3.3 (1.9–5.9)
Educational level of the mother				
Middle school or higher	70 (26.8)	191 (73.2)	1	1
Elementary school	251 (47.1)	283 (52.9)	2.4 (1.7–3.3)	1.8 (1.3–2.6)
No school	55 (56.1)	43 (43.9)	3.5 (2.1–5.7)	2.2 (1.3–3.8)

95% CI: 95% confidence interval; OR: odds ratio;

*: adjusted with household wealth index

In Morondava, a low level of maternal education and low socioeconomic status of the household were risk factors for low dietary diversity. In addition, the probability of having a low dietary diversity score was 1.8-fold (95% CI: 1.2–2.7) higher for children living in households that do not raise livestock than for those living in households that do (Table 3).

**Table 3:** Results of the multivariate analysis between the dietary diversity score and the explanatory variables, Morondava district: Final model.

	Dietary diversity score		Crude OR (95% CI)	Adjusted OR* (95% CI)
	Low N (%)	Adequate N (%)		
Educational level of the mother				
Middle school or higher	31 (23.3)	102 (76.7)	1	1
Elementary school	194 (44.6)	241 (55.4)	2.6 (1.7–4.1)	2.4 (1.5–6.4)
No school	219 (60.3)	144 (29.7)	5.0 (3.2–7.9)	4.0 (2.5–6.4)
Household socioeconomic level				
4^th^ Quartile (wealthiest)	73 (32.9)	149 (67.1)	1	1
3^rd^ Quartile	103 (46.8)	117 (53.2)	1.8 (1.2–2.6)	1.4 (1.0–2.1)
2^nd^ Quartile	119 (51.7)	111 (48.3)	2.2 (1.5–3.2)	1.5 (1.0–2.2)
1^st^ Quartile (poorest)	149 (57.5)	110 (42.5)	2.7 (2.0–4.0)	1.8 (1.2–2.8)
Possession of livestock				
Yes	365 (45.4)	439 (54.6)	1	1
No	79 (62.2)	48 (37.8)	2.0 (1.3–3.0)	1.8 (1.2–2.7)

95% CI: 95% confidence interval; OR: odds ratio;

*: adjusted with age

## Discussion

We performed a study at the HDSS of Moramanga and in the commune of Bemanonga in the Morondava district to describe the diet of children aged 6–59 months, including dietary diversity. Further statistical analysis allowed for the identification of determinants of a low dietary diversity score. Breastfeeding up to the age of 12 months was very common at both study sites, although almost half (46.5%) of the children in Moramanga and approximately three-quarters (76.0%) of those in Morondava did not benefit from early breastfeeding. The children's diet consisted mainly of starchy foods (based on cereals, roots, and tubers), seasonal fruits and vegetables. The proportion of children with a low dietary diversity score was higher in Morondava (47.6%) than in Moramanga (42.1%). At both study sites, a low level of education of mothers was risk factor for a low dietary diversity score. Children aged 6–11 months were more likely to have a relatively non varying diet than older children. In Morondava, not having farm animals and belonging to the poorest household wealth index was also risk factors.

Breastfeeding for up to 12 months was frequent. However, early breastfeeding was less frequent: only half the children of Moramanga and not even a quarter of the children of Morondava started breastfeeding early. The region of Menabe, which is part of the Morondava study site, is the region in Madagascar with the second-highest proportion of children who do not start to breastfeed early (40.7%)[9]. The WHO recommends breast-feeding within one hour of delivery, as colostrum is highly nutritious and contains antibodies to protect the child [14]. The poor perception of mothers of colostrum has been reported in the southern and southwestern regions of Madagascar, leading to calls for increased awareness and education of mothers (or influential individuals) on the benefits of colostrum [15,16]. Dietary diversity is an important element for appreciating the quality of the diet, although assessments are difficult in many developing countries. The methods used to estimate dietary intake require time and resources [17]. The diets of children in our two study sites were typical of those of developing countries: often poorly balanced; composed mainly of foods rich in carbohydrates, vegetables, and seasonal fruits; and poor in meat products [18]. Rice was the most highly consumed starchy food. Madagascar is among the countries with the highest rice consumption, which is estimated to be ≥ 300 g/d per inhabitant [19]. Consistent with findings from other studies [20,21], the dietary diversity score at our two study sites from the age of 12 months was higher than that of children aged 6 to 11 months. This may be because older children eat the foods prepared for the family as a whole and thus eat the same meals as other family members. In our two study sites, 56.0% of the children with data available on the type of dish consumed (n = 1003) consumed a family dish the day before. The proportion increased with child age: 43.1% for children aged 6–11 months, 49.8% for those aged 12–23 months, and approximately 60% for those aged 24 months.

The proportion of children with a low dietary diversity score was significantly higher in Morondava than in Moramanga. More than two in five children (47.6%) in Morondava had a low dietary diversity score, whereas in Moramanga, the proportion was approximately 42.1%. The difference may be because Morondava is in a region where the proportion of households with food insecurity is higher. A survey conducted in 2010 suggested that 41.1% of households in the Menabe region were food insecure, whereas the proportion was only 9.6% in the more highly developed Alaotra-Mangoro region [22].

We show that a low education level of mothers and low socioeconomic status of the child's household increased the likelihood of having a low dietary diversity score. The importance of the level of education of the parents, particularly the mother, on children's food practices has already been demonstrated in developing countries [21,23]. The quality of the diet of children during their early years depends mainly on the behavior and decisions of the mothers or those who usually care for the child [24]. Efforts should be made to improve the education of women and girls, as this could improve children's dietary practices, and particularly dietary diversity. In addition, nutrition education and awareness programs should be adapted to women and mothers with poor education. In Madagascar, the ratio of girls to boys in education decreases as the level of education increases: 1.05 for primary education, 0.8 for secondary education, and 0.7 for higher education [25]. One of the many reasons for extending the education of girls is that it later becomes a major determinant of their children's diets. Universal education, a complete cycle of free primary and secondary education by 2030 for girls and boys, constitutes Objective 4 of the Sustainable Development Objective (SDO) [26].

A low household socioeconomic status was a risk factor for child dietary diversity for children at the Morondava study site: the proportion of children with a low dietary diversity score increased with decreasing household socioeconomic score. Our results are consistent with those of other groups [20,23,27–29] and may reflect food insecurity in these households. Indeed, a lack of resources undoubtedly reduces the purchasing power, and thus, access to food, of households.

In Morondava, the risk of low dietary diversity scores was higher in households with no livestock than those with at least one animal (Adjusted OR: 1.8, 95% CI: 1.2–2.7). Ownership of livestock can directly improve the quality of children's diets if livestock products are used for home consumption (milk and milk products, eggs, meat)[30]. The income from livestock products can also indirectly contribute to the improvement of the children's diet, if appropriately managed [31,32]. Data on the use of livestock products or the origin of foods consumed by the households were not available in this study, and we found no statistically significant association between meat consumption by the child the day before the survey and the possession of livestock by the household. Indeed, one limitation of this study is that data on the children's diet were collected by single 24-hour recall; evidence suggests that the use of repeated, nonconsecutive 24-hour recall might improve data reliability and the estimation of diet [33]. However, we found some common determinants that were reported elsewhere, and the appropriate modeling for confounding effects adds strength to the validity of our findings. The lack of seasonality information could also be a limitation; a study conducted in Ethiopia with representative data suggested a seasonal variation in dietary diversity likely related to many factors (fasting period, lower availability of production) [34]. Studies including follow-up visits could examine seasonality more explicitly.

Our study shows that the diet of more than two-fifths of the children at our study sites in Bemanonga Commune, Morondava and the HDSS of Moramanga, were not diversified; this finding applied particularly to the youngest children (6–11 months). The low level of education of the mothers was the main risk factor for the low dietary diversity score in Moramanga. In Morondava, the low socioeconomic level of households and the lack of ownership of farm animals increased the likelihood of having a low dietary diversity score. One strength of this study is the random selection of the participants, such that they are representative of the children aged 6 to 59.9 months in the two zones. Few studies about infant and young child feeding practices have been conducted in Madagascar [6,29], and our results were complementary. Our study contributes to the understanding of the association between dietary diversity and its determinants. Our findings indicate that improving the level of education of women would be beneficial in the long term and that in the short term, nutrition education programs (messages transmitted, type of educational materials) should be adapted for women with a low educational level to improve children's food practices.

Interventions favoring food accessibility for disadvantaged households should be strengthened, including the promotion of husbandry practices and the use of the resulting food products [35]. Programs to reduce poverty and improve food availability through conditional transfer programs could be helpful [36]. We recommend programs that integrate several types of intervention (maternal education, food accessibility, poverty reduction), as they will improve children's dietary diversity and possibly their nutritional status.

## Supporting information

S1 Data Data used for the analysis. (XLSX)
